# Biased but in Doubt: Conflict and Decision Confidence

**DOI:** 10.1371/journal.pone.0015954

**Published:** 2011-01-25

**Authors:** Wim De Neys, Sofie Cromheeke, Magda Osman

**Affiliations:** 1 Centre National de la Recherche Scientifique (CNRS), University of Toulouse, Toulouse, France; 2 Department of Psychology, University of Leuven, Leuven, Belgium; 3 School of Biological and Chemical Sciences, Queen Mary University of London, London, United Kingdom; University of Leuven, Belgium

## Abstract

Human reasoning is often biased by intuitive heuristics. A central question is whether the bias results from a failure to detect that the intuitions conflict with traditional normative considerations or from a failure to discard the tempting intuitions. The present study addressed this unresolved debate by using people's decision confidence as a nonverbal index of conflict detection. Participants were asked to indicate how confident they were after solving classic base-rate (Experiment 1) and conjunction fallacy (Experiment 2) problems in which a cued intuitive response could be inconsistent or consistent with the traditional correct response. Results indicated that reasoners showed a clear confidence decrease when they gave an intuitive response that conflicted with the normative response. Contrary to popular belief, this establishes that people seem to acknowledge that their intuitive answers are not fully warranted. Experiment 3 established that younger reasoners did not yet show the confidence decrease, which points to the role of improved bias awareness in our reasoning development. Implications for the long standing debate on human rationality are discussed.

## Introduction

Human judgment is often biased by erroneous intuitions. Consider, for example, the success of the popular “Buy One, Get Second One 50% Off” sale you often see at retail stores. If you buy one item you get the opportunity to buy a second, similar one for only half of the original price. Even when we do not need the second item, we will often be tempted to buy it simply because our intuition is telling us that by not taking the offer we are missing out on a unique opportunity to get something for “only half of the original price”. From a normative point of view, however, this behavior is quite irrational. If you do not need a specific good, spending any money to obtain it is a waste of scarce financial resources. Hence, while we intuitively think that we are saving money, the store marketeers are actually tricking us to spend more than we should.

Decades of reasoning and decision-making research have shown that similar intuitive thinking is biasing people's judgment in a wide range of situations and tasks [1], [2]. In general, human reasoners seem to have a strong tendency to base their judgment on fast intuitive impressions rather than on more demanding, deliberative reasoning. Although this intuitive or so-called “heuristic” thinking might sometimes be useful, it will often cue responses that conflict with normative logical or probabilistic considerations and bias our decision-making.

Whereas it is well established that human judgment is often biased, the nature of this bias is far less clear. A central question is whether or not people know that they are biased and detect that their intuitive conclusions are not logically warranted. Some influential authors have argued that the widespread heuristic bias can be attributed to a failure to monitor our intuition [3]. Because of lax monitoring people would simply fail to detect that the intuitive response conflicts with logical considerations. However, others have suggested that there is nothing wrong with the detection process (e.g., [4]–[6]). According to these authors, people do notice that their intuitive response conflicts with traditional normative considerations. The problem, however, is that despite this knowledge they will not always manage to inhibit and discard the tempting intuitive beliefs. Thus, people “behave against their better judgment” [4] when they give an unwarranted heuristic response: They detect that they are biased but simply fail to block the biased response. In sum, according to this flawless detection view biased decisions are attributed to an inhibition failure rather than a conflict detection failure per se.

Clarifying the efficiency of the detection process and the nature of the heuristic bias is paramount for the development of reasoning and decision-making theories. The issue has also far-reaching implications for our view of human rationality (e.g., [7], [8]). Unfortunately, deciding between the two views has not been easy [9], [10]. Consistent with the lax detection view, it has long been established that reasoners' online verbalizations and retrospective response justifications do not indicate that they are taking any traditional logical or probabilistic considerations into account during reasoning (e.g., [11], [12]). For example, in one study De Neys and Glumicic [13] asked participants to think aloud while they were solving problems that were modelled after Kahneman and Tversky's [2] classic base-rate neglect problems. Consider the following example:

A psychologist wrote thumbnail descriptions of a sample of 1000 participants consisting of 995 females and 5 males. The description below was chosen at random from the 1000 available descriptions.

Jo is 23 years old and is finishing a degree in engineering. On Friday nights, Jo likes to go out cruising with friends while listening to loud music and drinking beer.

Which one of the following two statements is most likely?

- Jo is a man
- Jo is a woman

From a probabilistic point of view, given the size of the two groups in the sample, it will be more likely that a randomly drawn individual will be female (i.e., the largest group in the sample). However, intuitively many people will be tempted to respond that the individual is a male based on stereotypical beliefs cued by the description (“Jo is an engineer and drinks beer”).

The central question for De Neys and Glumicic [13] was whether verbal protocols would indicate that when people selected the intuitive response option (“a. Jo is a man”) they at least referred to the group size information during the reasoning process (e.g., “ … because Jo's drinking beer and loud I guess Jo'll be a guy, *although there were more women* …”). Such a basic sample size reference during the reasoning process can be considered as a minimal indication of successful bias detection: It indicates that people are not simply neglecting the normative base-rate information. Results clearly showed, however, that except for the few participants who gave the probabilistic base-rate response (“b. Jo is a woman), people hardly ever mentioned the base-rates. Hence, consistent with the lax detection view and numerous classic verbalisation studies, the explicit protocols suggested that biased reasoners are indeed mere intuitive thinkers who do not detect that their intuition conflicts with normative considerations.

Studies that started looking at more implicit detection measures, however, have presented support for the flawless detection view (e.g., [14]–[16]). For example, De Neys et al. [15] used fMRI to monitor the activation of a specific brain area, the anterior cingulate cortex (ACC), which is believed to mediate conflict detection during thinking. Participants again solved the classic base-rate problems in which the base-rates and personality description cued a conflicting response. Participants were also presented with no-conflict control versions in which the base-rates were switched around so that both the base-rates and description cued the same response. If people indeed neglected the base-rates, as the explicit protocols suggested, and did not detect that base-rates and description cued inconsistent responses, the conflict and control problems should not be processed any differently. Results showed, however, that the ACC was much more activated when people solved the classic conflict versions than when they solved the control versions without such conflicts. This increased activation was equally clear for correctly and incorrectly solved conflict problems. Hence, even when people were biased, the ACC seemed to signal the intrinsic conflict between the cued intuitive and base-rate response. Bluntly put, although people were not explicitly referring to the base-rate information, their brains did seem to pick up that their response was not consistent with it. Further work with the base-rate task and other logical reasoning problems showed that this increased ACC activation for biased responses is also accompanied by an increased autonomic activation [17], increased response decision-time [13], [18], [19], and altered accessibility of stored information that is associated with the cued logical/probabilistic and intuitive responses (e.g., [13], [14], [16]).

In sum, although it is clear that people do not explicitly detect that they are erring, available evidence suggests that they do seem to be sensitive to the presence of conflict between cued intuitive and normative logical or probabilistic principles at a more implicit level. The lack of explicitation has been explained by arguing that the neural conflict detection signal should be conceived as an implicit “gut” feeling. The signal would inform people that their intuition is not fully warranted but people would not always manage to verbalize the experience and explicitly label the logical principles that are being violated [16] (see also [20] for related suggestions). Although this hypothesis is not unreasonable, it faces a classic caveat. Without discarding the possible value of implicit processing, the lack of explicit evidence does open the possibility that the implicit conflict signal is a mere epiphenomenon. That is, the implicit conflict detection research clearly established that some part of our brain is sensitive to the presence of conflict in classic reasoning tasks. However, this does not necessarily imply that this conflict signal is also being used in the reasoning process. In other words, showing that the presence of conflict is detected does not suffice to argue that reasoners also “know” that their intuition is not warranted. Indeed, a critic might utter that the fact that despite the clear presence of a conflict signal people do not report experiencing a conflict and keep selecting the erroneous response, questions the value of this signal. Hence, what is needed to settle the bias debate is some minimal (nonverbal) indication that this signal is no mere epiphenomenon but has a functional impact on the reasoning process. This issue is the focus of the present study.

A straightforward way to assess the functional relevance of the implicit conflict signal is to examine people's decision confidence after they solve a reasoning problem. If the detection signal is not merely epiphenomenal, but actually informs people that their intuitive response is not fully warranted, people's decision confidence should be affected. That is, if people detect that they are biased but simply fail to verbalize the experience, we should at the very least expect to see that they do not show full confidence in their judgments.

Of course, people might never show full confidence and there might be myriad reason for why individuals differ in their confidence ratings (e.g., [21], [22]). Note, however, that our main research question does not concern people's absolute confidence level. As with the initial detection studies, in the present study we will present participants classic conflict problems and newly constructed no-conflict control problems. The only difference between the two types of problems is that cued intuitions conflict with traditional normative principles in the conflict versions while intuition and normative principles cue the same response in the no-conflict versions. The aim of the confidence contrast for the two types of problems is to help decide the detection debate. If detection of the intrinsic conflict on the classic versions is functional for the reasoning process and informs people that their intuitive response is questionable, participants should show lower confidence ratings after solving conflict problems as compared to no-conflict problems. If people do not detect the presence of conflict or the signal has no impact on the reasoning process, confidence ratings for the two types of problems should not differ.

We tested the confidence predictions in two initial experiments. In Experiment 1 people were presented with problems based on the classic base-rate task [2]. Experiment 2 tested the predictions with another well-studied reasoning task, the conjunction fallacy [23], to examine the generality of the findings. In Experiment 3 we tried to validate the findings by testing the performance of a population of reasoners who have been shown to have suboptimal conflict detection skills. Developmental studies in the cognitive control field have established that basic conflict monitoring abilities are not fully developed before late adolescence and young adulthood (e.g., [24]–[26]). Therefore, in Experiment 3 we presented our reasoning problems to a group of early and late adolescents and also asked them to rate their decision confidence. Given that conflict detection should be less efficient for young adolescents, we predict that any possible confidence decrease after solving conflict problems with adults or late adolescents should be absent (or less clear at least) in early adolescents.

## Methods

### Ethics statement

All experiments in this study were conducted in accordance with the Declaration of Helsinki and approved by the local ethics committee of the University of Leuven. Written informed consent was obtained from all participants (or their parent or guardian).

### Experiment 1: Base-rate task

#### Participants

A total of 247 undergraduates who were taking an introductory psychology course at the University of Leuven (Belgium) participated in return for course credit. Participants provided written informed consent and the study was approved by the local ethics committee of the University of Leuven.

#### Material

Participants solved a total of six base-rate problems. Three of these were classic conflict problems in which the description of the person was composed of common stereotypes of the smaller population group tested (i.e., the description and the base-rates conflicted). In the three no-conflict problems the description and the base-rates agreed.

Problems were based on a range of stereotypes (e.g., involving gender, age, nationality, see Appendix S1 for an overview). Descriptions were selected on the basis of an extensive pilot study [16]. Selected descriptions for the conflict and no-conflict problems moderately but consistently cued one of the two groups. This point is not trivial. For convenience, we label responses that are in line with the base-rates as correct answers. However, if reasoners adopt a formal Bayesian approach (e.g., [27]) and combine the base-rates with the diagnostic value of the description, this can lead to complications when the description is extremely diagnostic. For example, imagine that we have a sample of males and females and the description would state that the randomly drawn individual “gave birth to two children”. Now, by definition, no matter what the base-rates in the sample are, one would always need to conclude that the person is a woman. We limited the impact of this problem by only selecting descriptions that were judged to have moderate diagnostic value. Given these restrictions one may generally conclude that the response that is cued by the base-rates should be selected if participants manage to refrain from giving too much weight to the intuitive answer cued by the description.

To make sure that the contrast between conflict and no-conflict problems was not affected by the selected material, the descriptions for the conflict and no-conflict problems were completely crossed. That is, problems that were presented as conflict problems to one half of the participants were presented as no-conflict problems to the other half of the participants (and vice versa) by switching the base-rates around. The order of the two response options (‘a’ and ‘b’) was also counterbalanced. For half of the problems the correct response (i.e., the response consistent with the base-rates) was option ‘a’ whereas for the other half the second response option (‘b’) was the correct one.

Each problem was presented on a separate page in a booklet. After participants had solved a problem they found a rating scale ranging from 0% (completely unconfident) to 100% (completely confident) with 5% units (see Figure S1 for an example) and the following instructions on the next page:

Bellow you find a scale from 0% to 100%. Please indicate how confident you are that the answer you just gave was the right one. Circle the number that matches your feeling of confidence:

#### Procedure

Participants were tested at the same time during a regular course break. On the first page of the booklet they received the following general instructions:

In a big research project a number of studies were carried out where short personality descriptions of the participants were made. In every study there were participants from two population groups (e.g., carpenters and policemen). In each study one participant was drawn at random from the sample. You'll get to see the personality description of this randomly chosen participant. You'll also get information about the composition of the population groups tested in the study in question. You'll be asked to indicate to which population group the participant most likely belongs.

The six base-rate problems were presented in one of four pseudo-random orders. We made sure that half of the presented booklets started with a conflict problem, while the other half started with a no-conflict problem.

### Experiment 2: Conjunction fallacy task

In Experiment 2 we investigated the generality of our findings by testing the same hypotheses with a different reasoning task. Participants were presented with problems that were based on the classic conjunction-fallacy task (e.g., the “Linda-Problem”, see [23]). Together with the base-rate task, the conjunction fallacy is probably one of the most popular examples of the biasing impact of heuristics on people's decision-making. In the task people typically read a short personality sketch, for example, ‘Linda is 31 years old, single, outspoken, and very bright. She majored in philosophy. As a student, she was deeply concerned with issues of discrimination and social justice, and also participated in anti-nuclear demonstrations.’ Participants are then asked to rank statements according to their probability, for example ‘(A) Linda is a bank teller’, and ‘(B) Linda is a bank teller and is active in the feminist movement’.

The conjunction rule, the simplest and most fundamental law of probability [23], holds that the probability of a conjunction of two events cannot exceed that of either of its constituents (i.e., p(A&B)≤p(A), p(B)). Thus, there should always be more individuals that are simply bank tellers than individuals that are bank teller and in addition also active in the feminist movement. However, people typically violate the conjunction rule and intuitively conclude that statement B is more probable than statement A based on the intuitive match with the stereotypical description.

As in Experiment 1, we presented people with both the classic conflict versions and newly constructed no-conflict control problems. After each problem people were again asked to indicate their response confidence.

#### Participants

A total of 147 undergraduates who were taking an introductory psychology course at the University of Leuven (Belgium) participated in return for course credit. None had participated in Experiment 1. Participants provided written informed consent and the study was approved by the local ethics committee of the University of Leuven.

#### Material

Participants solved two conjunction problems each. In each problem participants first read a short personality description of a character (based on the classic “Linda” or “Bill” descriptions, see [23]). Next, they were given two statements about the character and were asked to indicate which one of the two was most probable. One statement always consisted of a conjunction of two characteristics (one characteristic that was likely given the description and one that was unlikely). The other statement contained only one of these characteristics. Consider the following example:

Bill is 34. He is intelligent, punctual but unimaginative and somewhat lifeless. In school, he was strong in mathematics but weak in social studies and humanities.

Which one of the following statements is most likely?

- Bill plays in a rock band for a hobby
- Bill is an accountant and plays in a rock band for a hobby

We manipulated the conflict nature of the problems by changing the content of the non-conjunctive statement. In the classic conflict versions we presented the unlikely characteristic (e.g., Bill plays in a rock band for a hobby) as the non-conjunctive statement (see example above). In the no-conflict versions we presented the likely characteristic (e.g., Bill is an accountant) as non-conjunctive statement (see example bellow):

Bill is 34. He is intelligent, punctual but unimaginative and somewhat lifeless. In school, he was strong in mathematics but weak in social studies and humanities.

Which one of the following statements is most likely?

- Bill is an accountant
- Bill is an accountant and plays in a rock band for a hobby

Intuitively, people will tend to select the statement that best fits with the stereotypical description (i.e., the most representative statement, see [23]). Clearly, the fit will be higher for the likely than the unlikely characteristic with the conjunctive statement falling in between. Normative considerations based on the conjunction rule always cue selection of the non-conjunctive statement. Hence, on our no-conflict problems both intuition and normative considerations will cue selection of the non-conjunctive response whereas people will be intuitively tempted to pick the conjunctive statement on the conflict problems.

Each participant solved one conflict and one no-conflict problem. To make sure that the content of the problems did not affect the findings we crossed the scenario content and conflict status. For half of the participants the conflict problem was based on the Bill scenario and the no-conflict problem on the Linda Scenario (and vice versa for the other half). As in Experiment 1, the order of the two response options (‘a’ and ‘b’) was also counterbalanced. For one of the problems the correct response (i.e., the non-conjunctive statement) was option ‘a’ whereas for the other problem the second response option (‘b’) was the correct one.

Each problem was presented on a separate page in a booklet and followed by the same confidence rating scale as in Experiment 1.

#### Procedure

Participants were tested at the same time during a regular course break. As in Experiment 1 we made sure that half of the presented booklets started with a conflict problem, while the other half started with a no-conflict problem. The scenario content of the first problem was also counterbalanced.

### Experiment 3: Developmental study

#### Participants

A total of 109 young (Mean age = 13.14 year, SE = .10) and 126 late (Mean age = 16.32, SE = .08) adolescents volunteered to participate. Young adolescents were recruited from a suburban middle school and the late adolescents were students at an associated high school. Informed consent was obtained from the participants' parents or guardian. The study was approved by the local ethics committee of the University of Leuven and the school boards.

#### Material

All participants were presented with one booklet with four base-rate problems and one booklet with four conjunction problems. Half of the problems in each booklet were conflict problems and the other half no-conflict control problems. Problems were constructed as in Experiment 1 and 2 with the same randomization procedures, instructions, and confidence rating scales. The only difference was the exact content of the problems. The materials were selected based on a pilot study [28] in which young and late adolescents rated the stereotypicality of a large number of descriptions. We made sure to select stereotypical descriptions and characteristics that were familiar for both age groups. A complete overview of all problems can be found in the Appendix S1.

#### Procedure

Participants were tested during a standard one-hour course break in which they remained in their classroom. Participants were presented with two booklets. Half of the participants started with the conjunction booklet and the other half with the base-rate booklet. Participants were given a five minute break after they finished solving the first booklet.

## Results

### Experiment 1: Base-rate task

#### Accuracy

The accuracy on the base-rate problems replicated the findings in previous studies (e.g., [13], [15]). Participants seemed to neglect the base-rate information and erred on the vast majority of the conflict problems. On average, only 20% (SE = 1.81) of these problems were solved correctly. Also, as expected, people had few difficulties when intuitive beliefs and base-rates pointed towards the same conclusion. Correct response rates on the no-conflict control problems reached 95% (SE = .83), F(1, 246) = 1443.54, p<.0001, η^2^_p_ = .85.

#### Response confidence

**Figure 1:**
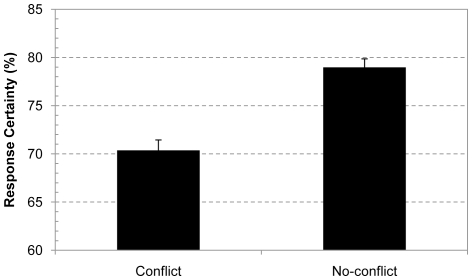
Response confidence for conflict and no-conflict base-rate problems. Average response confidence after solving conflict and no-conflict base-rate problems. Error bars are standard errors.

Our main question concerned people's decision confidence. If despite the poor performance on the conflict problems, people detect that their intuitive response conflicts with the base-rates, and know that their answer is questionable, then their confidence should be affected. As Figure 1 shows, overall confidence ratings were indeed about 10% lower for the classic conflict problems than for the control no-conflict problems, F(1, 246) = 54.98, p<.0001, η^2^_p_ = .18. Recall that the only difference between the conflict and no-conflict problems is the (in)consistency of the cued intuitive and base-rate response. If this intrinsic conflict was not detected or merely epiphenomenal, confidence ratings for the two types of problems should not have differed.

Although people typically erred on the conflict problems, on some occasions people did manage to give a correct response. A proponent of the bias-as-detection-failure view might therefore argue that it is those responses that are driving the overall confidence effect. Hence, it is still possible that there is no actual confidence effect for the intuitive responses. To eliminate such a possible confound we ran a separate analysis that was restricted to confidence ratings for incorrectly solved conflict problems. Results showed that we found the same 10% confidence decrease for the incorrectly solved conflict problems as in the overall analysis, F(1, 230) = 59.35, p<.0001, η^2^_p_ = .21. Note that in an additional control analysis we also made sure to remove the few trials in which the no-conflict problems were not solved correctly. However, as with all confidence analyses in the present study that took response accuracy into account, results were not shown to be affected by the elimination of these trials.

**Figure 2:**
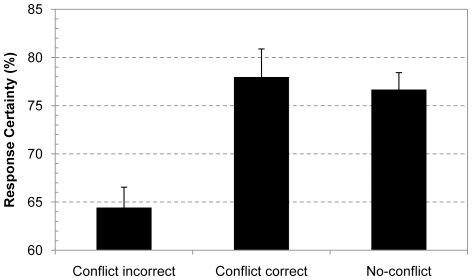
Response confidence for first-presented base-rate problem. Average response confidence for different types of responses on the first presented base-rate problem. Error bars are standard errors.

A second issue we need to address is the within-subject nature of the present conflict manipulation and the impact of possible learning effects. The initial studies that started focusing on conflict detection during thinking were typically quite lengthy. For example, in their fMRI study De Neys et al. [15] presented almost 100 problems. One might argue that the repeated presentation and repetitive nature of these studies cued participants to start paying attention to the conflict manipulation. Hence, the detection findings in these studies might simply be an artifact of learning. Note that we already reduced the number of presented items in the present study to limit the impact of such a learning confound. However, it has been argued that the purest test case in this respect concerns a between-subject experiment in which each subject solves only one single problem [29], [30]. To address this issue we ran an additional analysis in which we included only the confidence rating of the first problem that each participant solved (recall that this was a conflict problem for half of the participants and a no-conflict problem for the remaining half). As Figure 2 shows, results replicated the main finding of the overall analysis: The group of people who gave an intuitive response on the conflict problem were significantly less confident about their decision than the group of people who solved the no-conflict problem, F(1, 192) = 18.86, p<.0001, η^2^_p_ = .09. This establishes that the observed overall confidence decrease on the conflict problems does not result from a learning confound.

In the present study we were less concerned with confidence ratings for correctly solved conflict problems per se. The typical low accuracy rates on the conflict trials imply that the ratings for correctly solved conflict problems will be based on a small number of observations which might compromise the reliability of the data. Nevertheless, with this caution in mind, for completeness we also examined the confidence data of the group of people who gave the correct base-rate response on the crucial first conflict problem and included these in Figure 2. As the figure indicates, for people who solved the conflict problem correctly, confidence ratings did not seem to differ from the no-conflict ratings, F(1, 154)<1. This does suggest that good reasoners who reason in line with the normative standards also seem to know that they are right and show high response certainty. By itself this does not come as a surprise since after being confronted with the initial conflict these people manage to override the intuitive response and resolve the conflict. Nevertheless, as we noted, caution is needed when interpreting findings for the infrequent correct conflict responses. The main question in the present study concerns the confidence ratings for the common incorrect conflict responses. The decreased confidence on these problems compared to no-conflict control problems supports the claim that biased reasoners detect that their intuitive response on the classic conflict problems conflicts with the cued normative response.

### Experiment 2: Conjunction fallacy task

#### Accuracy

Participants' accuracy on the conjunction problems was as expected. In line with previous findings [23], the vast majority of participants committed the conjunction fallacy on the classic conflict problems. Correct response rates reached only 24% (SE = 3.5). However, as with the base-rate problems in Experiment 1, performance on the no-conflict control versions was much better with almost 96% (SE = 1.6) correct responses, Wilcoxon matched pairs test, n = 147, Z = 8.73, p<.0001.

#### Response confidence

**Figure 3:**
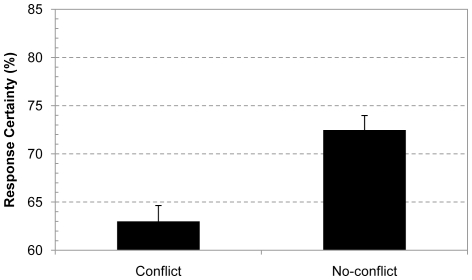
Response confidence for conflict and no-conflict conjunction problems. Average response confidence after solving conflict and no-conflict conjunction problems. Error bars are standard errors.

As Figure 3 shows, the confidence results nicely replicated the findings with the base-rate problems in Experiment 1. Despite the low accuracy, overall confidence ratings were again about 10% lower for the classic conflict problems than for the control no-conflict problems, F(1, 146) = 24.49, p<.0001, η^2^_p_ = .14. As in Experiment 1, this effect was equally clear when the analysis was restricted to incorrectly solved conflict trials, F(1, 106) = 13.72, p<.0005, η^2^_p_ = .12.

**Figure 4:**
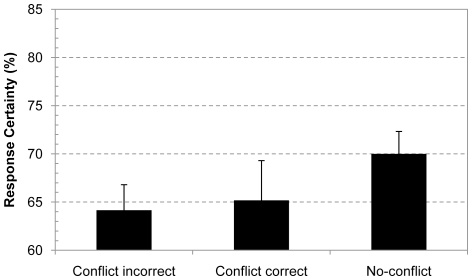
Response confidence for first-presented conjunction problem. Average response confidence for different types of responses on the first presented conjunction problem. Error bars are standard errors.

Finally, we also restricted the analysis to the first presented item and contrasted the confidence of the group of people who gave an incorrect conflict response and the confidence of people who solved a no-conflict control problem first. Figure 4 shows the results. Despite the smaller sample size the confidence effect was still marginally significant in this between-subject analysis, F(1, 120) = 2.85, p<.095, η^2^_p_ = .02. As in Experiment 1, the between-subject confidence contrast on the first item was not significant for the correctly solved conflict items, F(1, 89) = 1.77, p = .19.

### Experiment 3: Developmental study

Experiment 1 and 2 established that biased reasoners showed decreased confidence in their answers after solving conflict problems. Consistent with the flawless detection view, this suggests that biased reasoners at least acknowledge that their intuitive answer is questionable. In Experiment 3 we tried to validate the findings by testing the confidence contrast for conflict and no-conflict problems in a group of young and late adolescents. Given that elementary conflict monitoring skills are not fully developed before late adolescence (e.g., [25], [26]) we predicted that conflict detection during thinking will be less successful for the youngest reasoners. If young adolescents do not yet detect that their intuitive response conflicts with the cued normative response, they should not treat the conflict and no-conflict problems any differently and show similar confidence in their responses for both types of problems. Therefore, the decreased confidence after solving conflict problems should be far less pronounced for early than for late adolescents.

#### Accuracy

We ran a 2 (Conflict; conflict or no-conflict problem)×2 (Task; base-rate or conjunction task)×2 (Age Group; young or late adolescents) mixed model ANOVA on the mean accuracy scores. The first two factors were within-subjects factors and the Age Group was a between-subjects factor. Results showed that there was a main effect of the Conflict factor. Just as with the adults in Experiment 1 and 2, accuracy was near perfect on the no-conflict problems but significantly lower on the classic conflict problems, F(1, 233) = 2371.46, p<.0001, η^2^_p_ = .91. There was also a main effect of Age group, F(1, 233) = 8.03, p<.01, η^2^_p_ = .03, and the Age and Conflict factors interacted, F(1, 233) = 4.56, p<.05, η^2^_p_ = .02. Planned contrasts showed that age did not affect accuracy on the no-conflict problems, F(1, 233)<1, but performance on the conflict problems did increase slightly for late adolescents, F(1, 233) = 4.56, p<.05, η^2^_p_ = .02. However, despite the developmental increase even the oldest age group was typically biased with accuracies on the conflict problems below 20%.

The accuracy findings were very similar for the base-rate and conjunction problems. Neither the Task factor nor any of its interactions with the other factors reached significance. A complete overview of the accuracy findings can be found in Table 1.

**Table 1:** Overall accuracy and response confidence on the first item in two age groups.

		Base-rate task				Conjunction fallacy task			
		Young adolescents		Late Adolescents		Young adolescents		Late Adolescents	
Measure	Problem	Mean (SE)	n	Mean (SE)	n	Mean (SE)	n	Mean (SE)	n
Accuracy	Conflict	7 (2.5)	109	16 (2.3)	126	11 (2.8)	109	18 (2.7)	126
	No-conflict	97 (1.3)	109	95 (1.2)	126	92 (1.6)	109	96(1.5)	126
Confidence	Conflict incorrect	83 (4.2)	21	67 (4.0)	22	66 (3.7)	27	63 (3.5)	30
	No-conflict	82 (3.8)	25	82 (3.6)	28	73 (3.9)	30	75 (3.8)	35
	Conflict correct	59 (11.4)	4	73 (9.3)	6	55 (16.1)	2	46 (10.2)	5

#### Response confidence

**Figure 5:**
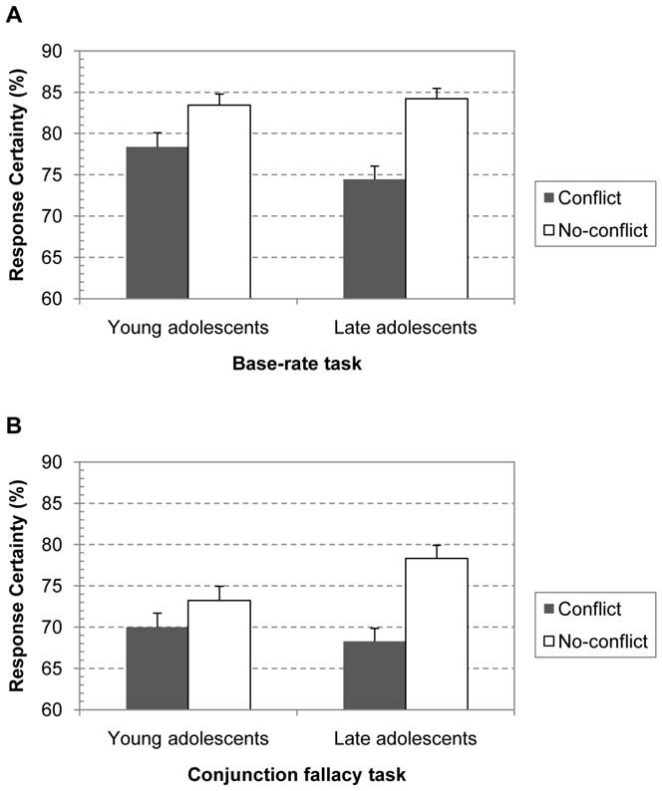
Response confidence in different age groups. Average response confidence after solving conflict and no-conflict base-rate (A) and conjunction (B) problems in the different age groups. Error bars are standard errors.

We also ran a 2 (Conflict; conflict or no-conflict problem)×2 (Task; base-rate or conjunction)×2 (Age Group; young or late adolescents) mixed model ANOVA on the mean confidence ratings. Figure 5 shows the results. There was a main effect of Conflict with overall lower confidence ratings for the conflict than for the no-conflict problems, F(1, 233) = 78.75, p<.0001, η^2^_p_ = .26. However, as predicted, this effect interacted with Age Group, F(1, 233) = 12.84, p<.0005, η^2^_p_ = .05. Although the conflict contrast was significant for both young, F(1, 233) = 13.05, p<.0005, η^2^_p_ = .05, and late adolescents, F(1, 233) = 83.64, p<.0001, η^2^_p_ = .26, the confidence decrease was much smaller in the youngest age group (i.e., 4% vs. 10%, t(233) = 3.58, p<.0005, d = .47). The main effect of Age Group was not significant, F(1, 233)<1.

There was also a main effect of the Task factor, F(1, 233) = 78.07, p<.0001, η^2^_p_ = .25. As Figure 5 shows, confidence ratings for the base-rate problems seemed to be overall higher than ratings for the conjunction problems. However, neither the higher-order interaction between the Task, Conflict and Age factors, F(1, 233)<1, nor any of the other interactions with the Task factor reached significance. As Experiment 1 and 2 already suggested, this establishes that the impact of conflict on the confidence measure is very similar in the two types of tasks. This consistency across reasoning tasks further supports the generality of the findings.

We also repeated the above analysis but made sure to exclude confidence ratings for correctly solved conflict trials. As in Experiment 1 and 2, the pattern remained unchanged. There was a significant main effect of the Conflict, F(1, 206) = 78.59, p<.0001, η^2^_p_ = .28, and Task, F(1, 206) = 55.77, p<.0001, η^2^_p_ = .21, factors. Once again, the conflict effect was less pronounced in the youngest age group, F(1, 206) = 9.07, p<.005, η^2^_p_ = .04. Other effects and interactions were not significant.

**Figure 6:**
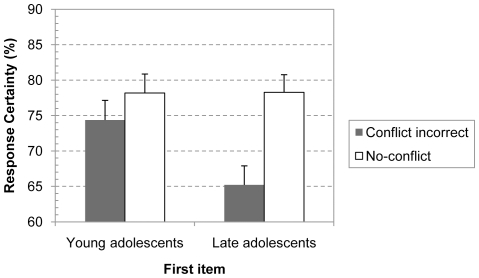
Response confidence for first problem in different age groups. Developmental impact on the response confidence of incorrect conflict responses on the first presented problem. Error bars are standard errors.

Finally, we also ran a between-subjects analysis on the confidence ratings for the first presented problem. The analysis focused on the contrast between the confidence ratings of the group of students who failed to solve the first conflict problem and those who solved a no-conflict problem (given that there were only six out of 109 young adolescents who responded correctly on the first presented conflict item we refrained from analyzing these confidence responses, see Table 1 for complete overview). The confidence data was entered in a 2 (Conflict; incorrect conflict or no-conflict problem)×2 (Task; base-rate or conjunction)×2 (Age Group; young or late adolescents) between-subjects ANOVA. The pattern for the first item was consistent with the overall analysis. There was a main effect of the Task, F(1, 201) = 10.53, p<.005, η^2^_p_ = .05, and Conflict factors, F(1, 201) = 10.02, p<.005, η^2^_p_ = .05, and the Conflict and Age Group factors tended to interact, F(1, 201) = 3.01, p<.085, η^2^_p_ = .02. Other effects and interactions were not significant. The Conflict×Age Group interaction is illustrated in Figure 6. Planned contrasts showed that the conflict contrast was significant for the oldest age group, F(1, 201) = 5.62, p<.025, η^2^_p_ = .03, but not for the young adolescents, F(1, 201)<1. Hence, on the first item confidence of young adolescents did not yet decrease when they gave a biased conflict response. This suggests that contrary to older reasoners, young adolescents do not yet detect that their intuitive response is unwarranted.

## Discussion

Consistent with decades of reasoning and decision making research, reasoning accuracies in the present study showed that people are typically biased and fail to select the normatively correct response on classic reasoning problems. However, our confidence measure indicated that despite this resounding bias, adults and older adolescents are detecting that their intuitive response is questionable. Three experiments established that reasoners' decision confidence on classic conflict problems was consistently lower than their confidence on the control no-conflict problems. The only difference between the conflict and no-conflict problems was that the cued intuitive response conflicted with traditional normative considerations on the classic versions. If reasoners were not detecting this conflict or the detection was merely epiphenomenal, their response confidence should not have decreased. This establishes that although people do typically not manage to discard a biased intuitive answer, they at least seem to be aware that their intuitive response is not fully warranted. Our developmental evidence in Experiment 3 suggested that it is precisely this bias awareness that younger reasoners lack.

The confidence findings help to clarify the nature of heuristic bias and validate the flawless detection view. We noted that although people hardly ever explicitly refer to normative considerations during reasoning, more implicit detection measures such as the activation of the anterior cingulate cortex or autonomic skin-conductance levels already indicated that our brain is sensitive to the presence of conflict between cued intuitive and normative considerations (e.g., [15], [17]). The present findings establish that this detection signal is not epiphenomenal. Giving an intuitive response that conflicts with more normative considerations does not simply result in some fancy brain-activation but directly affects our judgment. People literally indicate that their intuitive response is not fully warranted. Clearly, the well-established lack of explicit verbalization suggests that this knowledge is implicit in nature. People will not manage to label and identify the exact normative principles that are being violated. However, whenever their intuitive answer conflicts with more normative considerations they do seem to acknowledge that their response is questionable. The fact that this conflict is affecting their judgment implies that reasoners at least implicitly adhere to these normative principles.

At a more general level our findings help to sketch a more optimistic view of human rationality. Note that over the decades, the continuous confrontation with the strikingly low accuracy of educated adults on the classic reasoning tasks has led researchers to question human rationality and traditional normative standards [8], [31]. In a nutshell, some researcher argued that the widespread bias implied that humans are illogical and irrational intuitive reasoners (e.g., [32], [33]). Others argued that the low accuracy pointed to the invalidity of the traditional logical or probabilistic normative rules [34]–[36]. According to this latter view, humans are adhering to other norms than the traditional normative logical standards when solving classic reasoning tasks. People would interpret tasks such as the base-rate or conjunction fallacy task as a type of social classification problem in which they try to determine to which social group a character belongs. Given this alternative task interpretation the intuitive response would be perfectly valid. These issues have resulted in a debate that has raged through the field for decades without clear solution [8]. The present findings shed light on this and support a conclusion that might help to save human rationality and the traditional normative standards: The lower confidence implies that people are at least implicitly taking the normative principles into account when solving the classic conflict problems. If adult reasoners would not master the normative principles or would not consider these to be relevant, there would be nothing to conflict with their responses, and so people's response confidence should not be affected. It has previously been argued that the whole rationality discussion in the reasoning field has been biased by an almost exclusive focus on accuracy rates and the output of the reasoning process (e.g., [37]–[40]). The present work underscores this point and indicates that if we scratch below the accuracy surface, people are more normative than their biased responses suggest.

This being said, it is important to address some potential critiques with respect to our study. As we stated, our findings imply that people show some minimal sensitivity to base-rates and the conjunction rule in classic reasoning tasks. One might wonder whether this point has been demonstrated in past studies. It is true that a number of manipulations and interventions (e.g., making base-rates more extreme or making the description less diagnostic) have been shown to increase people's reasoning accuracy (e.g., [41]). This indicates that it is possible to have people select the correct response and take base-rates into account, for example. However, that is not the issue here. The question is: Are people taking the base-rates and conjunction rule into account when they give an intuitive response? This question cannot be answered by looking at accuracy rates per se. Indeed, even if, for example, people show perfect accuracy when the base-rates are made more extreme, this can never establish whether or not they were taken into account initially. This is precisely the reason why the diametric accounts on conflict detection persist in the reasoning and decision-making literature. The present confidence data and study design are critical to address this question.

We do believe that there is an interesting link between the present findings and an earlier study on metacognitive uncertainty during syllogistic reasoning by Quayle and Ball [42]. These authors observed that although people often judged invalid syllogistic conclusions to be valid, their subjective confidence ratings for these erroneous judgments were typically lower than for valid problems. Although Quayle and Ball did not manipulate the conflict nature of their problems, the results do seem to fit with the basic idea that people are sensitive to normative violations and might be more logical than their erroneous responses suggest. This strengthens the generality of our claims with respect to the validity of traditional normative standards.

In our work we have been specifically contrasting the lax and flawless views on conflict detection during thinking. We noted that the present confidence findings are consistent with the flawless detection view. However, one might want to consider alternative conceptualizations. For example, the present findings also fit with a “weighing view”. The idea behind the weighing view is that people are simply weighing competing arguments when solving the conflict problems. People would consider the normative response on the conflict problems, find it unpersuasive or weaker than the intuitive response and therefore go with the intuitive response. The flawless detection view entails that people notice that their intuitive response conflicts with the normative response, try to block it but fail to do so because of the compelling nature of the intuitive response. The weighing view also entails that people experience a conflict, but suggests that precisely because people find the intuitive response so compelling, they simply see no need to engage in an inhibition process. Hence, the difference between the two views lies in the postulation of an additional inhibition process.

It is important to stress that the flawless detection and weighing views make similar claims with respect to reasoners' conflict sensitivity and subjective knowledge state. Note that the flawless detection view does not entail that biased reasoners are 100% convinced that the normative response is correct. The whole point is that people will be in doubt. If people detect the conflict and this has any functional impact on their reasoning process, they should show decreased response confidence for intuitive responses on the conflict (vs. no-conflict) problems. This implies that the normative considerations have a minimal impact on people's judgment. Hence, it does not necessarily need to be the case that people consider the intuitive response less appropriate than the normative response per se. The point is that reasoners consider the intuitive response less compelling than the intuitive response on the no-conflict problems. In this respect the flawless detection and weighing view are consistent and point to the same implications: If reasoners decide after weighing to go with the intuitive response, the weighing at least implies that the normative information has been given some minimal consideration. If people would find the normative response on the conflict problems completely unconvincing, their response confidence should not be affected.

For completeness, one might note that the postulation of an additional inhibition process has gained some credence from recent findings. For example, De Neys and Franssens [14], probed memory activations after reasoning to examine the inhibition process. In their study participants solved conflict and no-conflict versions of the base-rate problems (and related syllogistic reasoning problems). After each problem participants were presented with a lexical decision task in which they had to judge whether a presented letter string was a word or not. Half of the presented words were strongly associated with the intuitive response that was cued in the reasoning problem. Results showed that lexical decision times for these target words were longer after solving conflict vs. no-conflict problems. This classic inhibition effect was less pronounced but still significant when people gave the intuitive response on the conflict problems. This seems to argue against a mere weighing view. If people were not at least engaging in an inhibition process and tried to discard the intuitive response it becomes harder to explain why words that are closely associated with the cued intuition become less accessible in memory after the reasoning task (i.e., less accessible than after solving no-conflict problems). Nevertheless, it should be clear that with respect to reasoners' subjective knowledge state the flawless detection view is consistent with a weighing view. Both views entail that the intuitive response should be less compelling on conflict problems than when it does not conflict with normative considerations on the no-conflict problems. This implies that even when people give an intuitive response on the classic conflict problems they give some minimal weight to normative considerations such as the conjunction rule or the role of the base-rates. It is this critical norm sensitivity that the present confidence data establish.

We stated that our present confidence findings fit with the early flawless detection claims by Sloman and Epstein [4], [6], [43]. It should be noted, however, that the claims of these authors were rooted in specific dual process models of reasoning. For example, Sloman [6] has suggested that people will detect conflicts because they always simultaneously engage in more automatic intuitive processing and demanding analytic-logical processing. One implication of this view is that the detection is assumed to result from time-consuming and resource demanding analytic computations. For completeness, we should stress that we do not subscribe to these further dual process assumptions. The present confidence findings imply that people are taking traditional normative principles into account when solving the classic conflict problems. However, there is no need to assume that the activation of these principles itself is especially demanding in cognitive terms. We have pointed to a number of theoretical paradoxes associated with this assumption [13] (see also [44]), and have provided empirical data that indicates that the detection process is indeed quite effortless [16]. The interested reader can find an extensive discussion of the implications of our findings for dual process theories in De Neys and Glumicic [13]. The basic point we want to note here is that while we agree with Sloman and Epstein that detection is flawless, we do not necessarily share their specific dual process assumptions as put forward in their original models.

To avoid possible misinterpretations it is perhaps also informative to underline that our claims with respect to the norm validity are situated at the psychological processing level. Our study indicates that people are sensitive to violations of traditional norms during thinking. As we explained, this finding argues against the claim that people consider these traditional norms to be irrelevant for their judgment. However, clearly, the fact that people adhere to a certain norm does not by itself entail that the norm is valid. From an epistemological/philosophical point of view, it might still be that other norms are more appropriate. In other words, our claim with respect to the validity of traditional norms does not entail that these norms are ultimately correct, but rather that human reasoners *consider* them to be correct. It is this demonstrated adherence to the traditional normative principles that is crucial to counter the idea that people do not know these principles or do not consider them relevant to solve classic reasoning problems.

Finally, we would like to highlight that the present study might have interesting implications for the developmental field. Just as with the debate on human rationality, the apparent omnipresence of intuitive bias resulted in quite pessimistic developmental views. As Markovits and Barrouillet [45] noted, the demonstration of the widespread bias in human reasoning since the 1960s seemed to point to a developmental standstill in human reasoning (see [46] for studies criticizing this idea). In other words, if the vast majority of educated university students fail to solve basic reasoning problems, there surely does not seem to be a lot of development going on. At first sight, our developmental study might have seem to strengthen this conclusion. Although there was a slight performance increase when contrasting early and late adolescents' accuracy rates, even in late adolescence accuracy was only proximately 15%. However, looking closely at the conflict detection process and the confidence data suggests that the lack of development is more apparent than real. Although both adults and adolescents are indeed biased most of the time, our findings indicate that a possible important difference between the age groups is that adults at least detect that their responses are biased. Consistent with recent insights in the developmental field (e.g., [39], [46]–[48]) this differential bias awareness argues against the idea of a developmental standstill in human reasoning. Nevertheless, our developmental findings will need further validation. For example, although our confidence measure allowed us to document the differential bias awareness, it is not clear whether younger adolescents also lack the implicit neuronal conflict signal or merely its translation into a decreased response confidence (i.e., it might be that younger adolescents also showed implicit conflict-related brain activity but this activity might still be epiphenomenal). Clearly, directly studying the conflict-related brain activity of younger reasoners in an fMRI study would be very useful in this respect. Likewise, it would be interesting to further clarify whether the lack of conflict awareness primarily results from limited basic conflict monitoring skills per se or whether it is also affected by a possible less developed normative knowledge (e.g., see [28]). These outstanding questions will need to be addressed in more focused and fine-grained developmental studies.

In sum, the present paper indicated that although human reasoners might typically fail to refrain from giving biased responses, they do seem to acknowledge that their intuitive responses are not fully warranted. This implies that at least by the end of adolescence, human reasoners are more sensitive to normative standards than the historical omnipresence of the intuitive response bias suggests.

## Supporting Information

Appendix S1 **Overview of the problem content in Experiments 1 and 3.**
(DOC)

Figure S1 **Example of the confidence rating scale**
(TIF)
