# Correction: Biased but in Doubt: Conflict and Decision Confidence

**DOI:** 10.1371/annotation/1ebd8050-5513-426f-8399-201773755683

**Published:** 2011-02-09

**Authors:** Wim De Neys, Sofie Cromheeke, Magda Osman

The wrong email is listed for the corresponding author. The correct email address is: m.osman@qmul.ac.uk

