# Supplementary material for: Biased but in Doubt: Conflict and Decision Confidence
Source: PLoS One. 2011 Jan 25;6(1):e15954. doi: 10.1371/journal.pone.0015954 (PMC3026795; doi:10.1371/journal.pone.0015954)
Supplement: Appendix S1 — Overview of the problem content in Experiments 1 and 3. (DOC) [file pone.0015954.s001.doc]

**APPENDIX S1**

Overview of the problem content in Experiment 1:

1.

In a study 1000 people were tested. Among the participants there were 5 people who drive a used Nissan and 995 people who drive a BMW. Etienne is a randomly chosen participant of the study.

Etienne is 38. He works in a steel plant. He lives in a small apartment in the outskirts of Charleroi. His wife has left him.

What is most likely?

Etienne drives a used Nissan

Etienne drives a BMW

2.

In a study 1000 people were tested. Among the participants there were 5 sixteen-year-olds and 995 forty-year-olds. Els is a randomly chosen participant of the study.

Els likes to listen to techno and electro music. She often wears tight sweaters and jeans. She loves to dance and has a small nose piercing.

What is most likely?

Els is sixteen

Els is forty

3.

In a study 1000 people were tested. Among the participants there were 5 women and 995 men. Dominique is a randomly chosen participant of the study.

Dominique is a self-confident and competitive person. Dominique’s goal is building a career. Dominique does a lot of sport and is well-muscled.

What is most likely?

Dominique is a woman

Dominique is a man

4.

In a study 1000 people were tested. Among the participants there were 5 people who vote for the green party and 995 people who vote for the Flemish Interest party. Jeanine is a randomly chosen participant of the study.

Jeanine is 67. She worked as an assembly line packer. She believes that traditional values are important and lives in a high crime area.

What is most likely?

Jeanine votes for the green party

Jeanine votes for Flemish Interest

** Note: Flemish Interest is a conservative, anti-immigrant, far right party.*

5.

In a study 1000 people were tested. Among the participants there were 5 people who like to watch Canvas and 995 people who like to watch VTM. Aline is a randomly chosen participant of the study.

Aline is 35. She writes reviews for a magazine. Her husband works at the university. She loves painting and photography.

What is most likely?

Aline likes to watch Canvas

Aline likes to watch VTM

**Note:* *VTM is a popular, commercial (“Fox”-like) Flemish TV channel. Canvas is a more educational, publicly-funded (“PBS”-like) channel.*

6.

In a study 1000 people were tested. Among the participants there were 5 Swedes and 995 Italians. Mario is a randomly chosen participant of the study.

Mario is 25. He is a charming young man and is a real womanizer. His favourite dish is the spaghetti his mother makes.

What is most likely?

Mario is a Swede

Mario is an Italian

Overview of the problem content in Experiment 3:

*Base-rate problems*

1.

In a study 1000 people were tested. Among the participants there were 5 people who lived in the city and 995 who lived in the country. Allison is a randomly chosen participant of the study.

Allison is 22. She likes horseback riding and takes care of her pets after school. In the weekend she likes to rise early and visit her grandparents.

What is most likely?

Allison lives in the city

Allison lives in the country

2.

In a study 1000 people were tested. Among the participants there were 5 seventeen-year olds and 995 sixty-year olds. Anne is a randomly chosen participant of the study.

Anne is in good health. She is religious and goes to church every week. She is fond of knitting sweaters, gardening, and likes to gossip with her neighbors.

What is most likely?

Anne is seventeen

Anne is sixty

3.

In a study 1000 people were tested. Among the participants there were 5 twenty-year olds and 995 sixty-year olds. Karl is a randomly chosen participant of the study.

Karl likes to listen to rock music and goes to concerts. He painted his cell phone in the colors of his favorite football team.

What is most likely?

Karl is twenty

Karl is sixty

4.

In a study 1000 people were tested. Among the participants there were 5 people who drive a used Nissan and 995 people who drive a BMW. Etienne is a randomly chosen participant of the study.

Etienne is 38. He works in a steel plant. He lives in a small apartment in the outskirts of Charleroi. His wife has left him.

What is most likely?

Etienne drives a used Nissan.

Etienne drives a BMW.

*Conjunction problems*

1.

Redinant is 16. He wears old-fashioned clothes. He studies very well and is the teachers’ pet. He doesn’t have many friends and doesn’t do well with girls.

Which statement is most likely?

Redinant is often going to parties

Redinant is often going to parties and is being bullied

2.

James is 26. He lives in Knokke. He likes to wear designer clothes and acts somewhat stuck-up. On Sunday he plays golf with his father.

Which statement is most likely?

James volunteers in the day care center in free time

James volunteers in the day care center in free time and works as a stock broker

3.

Jake is 20. He grew up in a poor family in a neglected neighborhood. He is quite violent and already served a short sentence in prison.

Which statement is most likely?

Jake plays the violin

Jake plays the violin and is jobless

4.

Jon is 32. He is intelligent, punctual but unimaginative and somewhat lifeless. In school, he was strong in mathematics but weak in languages and art.

Which one of the following statements is most likely?

Jon plays in a rock band

Jon plays in a rock band and is an accountant
