# Supplementary figures and images for: Biased but in Doubt: Conflict and Decision Confidence
Source: PLoS One. 2011 Jan 25;6(1):e15954. doi: 10.1371/journal.pone.0015954 (PMC3026795; doi:10.1371/journal.pone.0015954)

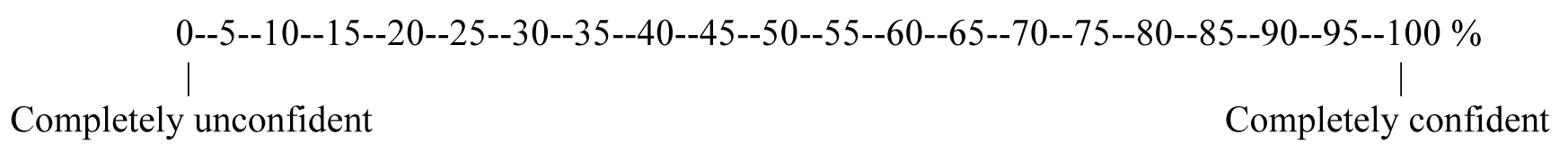

Supplement: Figure S1 — Example of the confidence rating scale (TIF) [file pone.0015954.s002.tif]
